# Testosterone and Anterior Cruciate Ligament Injury and Reconstruction Outcomes: A Systematic Review and Meta-analysis

**DOI:** 10.1177/19417381261486660

**Published:** 2026-10-15

**Authors:** Dave Osinachukwu Duru, David Kwan Ryung Lee, Prushoth Vivekanantha, Marc Daniel Bouchard, Osama Alzobi, Loiy Alkhatib, Bashir A. Zikria, Ashraf Hantouly, Moin Khan

**Affiliations:** School of Clinical Medicine, University of Cambridge, Cambridge, UK; School of Clinical Medicine, University of Cambridge, Cambridge, UK; Division of Orthopedic Surgery, McMaster University, Hamilton, Ontario, Canada; Division of Orthopedic Surgery, McMaster University, Hamilton, Ontario, Canada; Department of Orthopedic Surgery NYU Langone Health, New York, New York; Division of Orthopedic Surgery, Dr. Sulaiman Al Habib Hospital, Dubai, United Arab Emirates; Department of Orthopaedic Surgery, Johns Hopkins School of Medicine, Baltimore, Maryland; Division of Orthopedic Surgery, McMaster University, Hamilton, Ontario, Canada; Division of Orthopedic Surgery, McMaster University, Hamilton, Ontario, Canada

**Keywords:** ACL reconstruction, anterior cruciate ligament, risk factors, testosterone, testosterone replacement therapy

## Abstract

**Context:**

Testosterone influences connective tissue biology, neuromuscular performance, and postoperative recovery. However, its clinical impact on anterior cruciate ligament (ACL) injury risk and outcomes after ACL reconstruction (ACLR) remains unclear.

**Objective:**

To evaluate the association between testosterone replacement therapy (TRT) or endogenous testosterone levels and ACL tear incidence, ACLR revision, patient-reported outcome measures (PROMs), strength, and complications.

**Data Sources:**

Embase, Ovid MEDLINE, and PubMed were searched from inception to March 14, 2026.

**Study Selection:**

Studies evaluating TRT use or endogenous testosterone levels in relation to ACL tear incidence or outcomes after ACLR were included. Systematic reviews, narrative reviews, scoping reviews, letters, editorials, technical notes, conference abstracts without full-text publication, animal studies, cadaveric studies, case reports, and non-English studies were excluded.

**Study Design:**

Systematic review and meta-analysis.

**Level of Evidence:**

Level 3.

**Data Extraction:**

Two reviewers independently extracted study characteristics, testosterone exposure, ACL tear incidence, ACLR revision, PROMs, strength outcomes, and complications. Random-effects meta-analyses pooled odds ratios for ACL tear incidence and ACLR revision. Study quality was assessed using the Methodological Index for Non-Randomized Studies and Cochrane Risk of Bias 2.0, and certainty of evidence was assessed using Grading of Recommendations, Assessment, Development and Evaluation.

**Results:**

Seven studies were eligible, comprising 1,178,891 patients, of whom 589,436 were TRT users. TRT use was associated with a significantly increased risk of primary ACL tear within 2 years (odds ratio [OR], 2.83; 95% CI, 1.74-4.62; *I*^ 2^ = 95.4%), with consistent findings across sex-specific analyses. In contrast, there was no difference in ACLR revision rates at 2 years (OR 1.04; 95% CI 0.84-1.29; *I*^ 2^ = 0%). One randomized trial demonstrated increased early postoperative lean mass with TRT, without differences in strength or PROMs. Increased ACL rupture risk was associated with higher testosterone levels in men, and lower levels in women. Higher testosterone levels did not adversely affect PROMs or complication rates after ACLR.

**Conclusion:**

TRT is associated with increased ACL tear risk at 2 years after initiation but does not appear to affect 2-year ACLR revision rates. Higher endogenous testosterone levels may influence ACL injury susceptibility in a sex-dependent manner, without impairing ACLR recovery.

Anterior cruciate ligament (ACL) injuries account for 20% of athletic knee injuries,^16^ particularly affecting young active people participating in pivoting sports such as basketball and football.^5^ ACL injury, constituting both primary tears and graft failure after ACL reconstruction (ACLR), remains a significant clinical challenge. Consequently, increasing attention has been directed toward identifying biological risk factors that may influence ACL injury susceptibility.

Testosterone has emerged as a potential modulator of ACL injury risk.^29,33^ In recent years, interest in this hormone has increased due to rising adoption of testosterone replacement therapy (TRT) in aging populations to treat hypogonadism.^6^ Although TRT has benefits, supraphysiological exposure is associated with adverse effects, such as erythrocytosis, venous thromboembolism, and potential cardiovascular complications.^2,20^ In addition, excessive TRT exposure is associated with increased musculoskeletal injury risk,^18,23,30^ including ACL tears. Mechanistically, testosterone may increase ACL stiffness and disrupt extracellular-matrix remodeling, including through altered matrix metalloproteinase activity under repetitive loading.^9,17,24^ Conversely, aromatization of testosterone to estrogen may reduce lysyl oxidase-mediated collagen cross-linking and impair ligament mechanical properties.^14^ However, these mechanisms are derived predominantly from animal and in vitro studies.

Accordingly, the relationship specifically between testosterone and human ACL injury susceptibility remains incompletely understood. This question is increasingly clinically relevant. Not only does TRT use continue to rise, but exogenous androgen use is also increasingly prevalent among athletes seeking performance enhancement,^13^ given that higher testosterone levels are consistently linked to improved athletic ability.^4,12^ Simultaneously, emerging perioperative strategies are exploring anabolic hormonal optimization to improve recovery after ACLR.^19,33^ Whether such interventions confer net benefit or inadvertently increase ACL injury risk remains unclear.

To date, no systematic review has comprehensively synthesized the evidence examining the association between TRT use or endogenous testosterone levels and ACL injury, including both primary tear incidence and outcomes after ACLR. A thorough evaluation of the literature is therefore required to clarify the potential role of testosterone in ACL injury. The purpose of this systematic review was to evaluate the association between testosterone (TRT use or endogenous testosterone levels) and both (1) ACL tear incidence and (2) patient-reported outcome measures (PROMs) or complications after ACLR. The hypotheses were that (1) testosterone is associated with an increased risk of primary ACL tears and (2) testosterone may improve PROMs, without affecting revision risk, following ACLR.

## Methods

### Study Design

This systematic review was conducted in accordance with the Preferred Reporting Items for Systematic Reviews and Meta-Analyses (PRISMA) 2020 guidelines and the methodology was registered prospectively with PROSPERO (CRD420251242324).

### Search Strategy and Study Selection

Articles were searched in Ovid Embase, Ovid MEDLINE, and PubMed from database inception to March 14, 2026 by 2 independent reviewers. The search strategy combined controlled vocabulary and free-text terms related to testosterone (TRT and/or endogenous testosterone levels) and (1) primary ACL tear incidence or (2) PROMs, strength outcomes, and complications after ACLR. Medical Subject Headings and keywords included (“testosterone” or “hypogonadism” or “testosterone deficiency” or “testosterone replacement” or “testosterone replacement therapy” or “TRT” or “dihydrotestosterone” or “DHT” or “anabolic steroid”) and (“anterior cruciate ligament” or “ACL rupture” or “ACL tear*” or “ACL injur*” or “anterior cruciate ligament injur*” or “anterior cruciate ligament rupture” or “anterior cruciate ligament tear*” or “ACL reconstruction” or “ACLR”) (Supplementary Table 1, available in the online version of this article). The discrepancies between the reviewers were resolved through discussion or consultation with a senior author. Reference lists for all included studies were screened manually to identify eligible studies.

No date limits were applied. All search results were imported into Rayyan, and duplicates were removed. Two reviewers then screened titles and abstracts against the inclusion criteria. Two reviewers examined full-text articles to determine eligible studies for final inclusion, with the reasons for exclusion documented. Disagreements were resolved by discussion or consultation with a third reviewer.

### Eligibility Criteria

This review included studies that evaluated the effect of TRT and/or endogenous testosterone levels on susceptibility to ACL tears or PROMs, strength, and complications (e.g., revision surgery) after ACLR.

Exclusion criteria included systematic review articles, narrative reviews, scoping reviews, letters, editorials, technical notes, conference abstracts lacking full-text publication, animal studies, cadaveric studies, case reports, and studies not published in English.

### Data Extraction

Data were extracted independently by 2 reviewers using a predefined data collection form developed in Microsoft Excel (Version 16.100). The extracted variables included study characteristics such as author, year of publication, country, study design, and data source; whether ACL tear incidence and/or ACLR outcomes were investigated; details of TRT exposure, including the timing relative to surgery and duration, where reported. Primary outcomes were ACL tear incidence and revision after ACLR. Secondary outcomes included functional and pain PROMs, lean muscle mass, extensor strength outcomes, and nonsurgical complications.

### Methodological Quality Assessment

The quality of included studies was assessed by 2 reviewers via 2 tools based on the study design. Randomized controlled trials (RCTs) were evaluated using the Cochrane Risk of Bias 2.0 (RoB2) tool, which assesses risk of bias across 5 domains: randomization process, deviations from intended interventions, missing outcome data, measurement of the outcome, and selection of the reported result. Each of the domains was rated as low risk, some concerns, or high risk, with an overall risk of bias judgement assigned accordingly.^8^

Nonrandomized studies were assessed using the Methodological Index for Non-Randomized Studies (MINORS) criteria. Each study was scored across the MINORS domains, with a maximum total score of 16 for noncomparative and 24 for comparative studies.^25^ In this review, noncomparative studies were classified as poor (≤8), moderate (9-13), or good (14-16) quality, while comparative studies were classified as poor (≤14), moderate (15-21), or good (22-24) quality. Discrepancies in scoring were resolved through consensus or consultation with a third author.

### Data Synthesis and Analysis

In the primary analysis, statistical analysis was performed using R (Version 4.5.1) with the metafor package. Restricted maximum likelihood (REML) random-effects meta-analysis was conducted to estimate pooled effect sizes for ACL tear incidence and ACLR revision with versus without TRT use. A random-effects model was selected a priori due to anticipated methodological heterogeneity, including differences in follow-up duration, patient comorbidity profiles, and surgical indications. In accordance with previous recommendations, a minimum of 2 studies were included in each meta-analysis.^10,11^ Pooled unadjusted odds ratios (ORs) with 95% CI were calculated. Pooled estimates were derived as weighted averages of individual study effects, with weights reflecting inverse variance. Statistical heterogeneity was assessed using Higgins’ *I*^ 2^ statistic. *I*^ 2^ values of <50% were considered low heterogeneity, 50% to 75% reflected moderate heterogeneity, and >75% equated to high heterogeneity. Statistical significance was defined as *P* < 0.05, and pooled estimates were considered nonsignificant if the 95% CI crossed the null value. Where meta-analysis was not performed, findings were synthesized narratively. For the secondary narrative review on the association between endogenous testosterone levels and ACL tear incidence or ACLR PROMs/strength/complications, data were synthesized narratively.

## Results

### Search Results

A total of 230 records were identified through database searching. After removal of 92 duplicates, 138 studies underwent title and abstract screening, of which 128 were excluded; 10 articles were retrieved for full-text screening. From these 10 studies, 7 met the inclusion criteria and were included in the final analysis (Figure 1 and Table 1).

**Figure 1.**
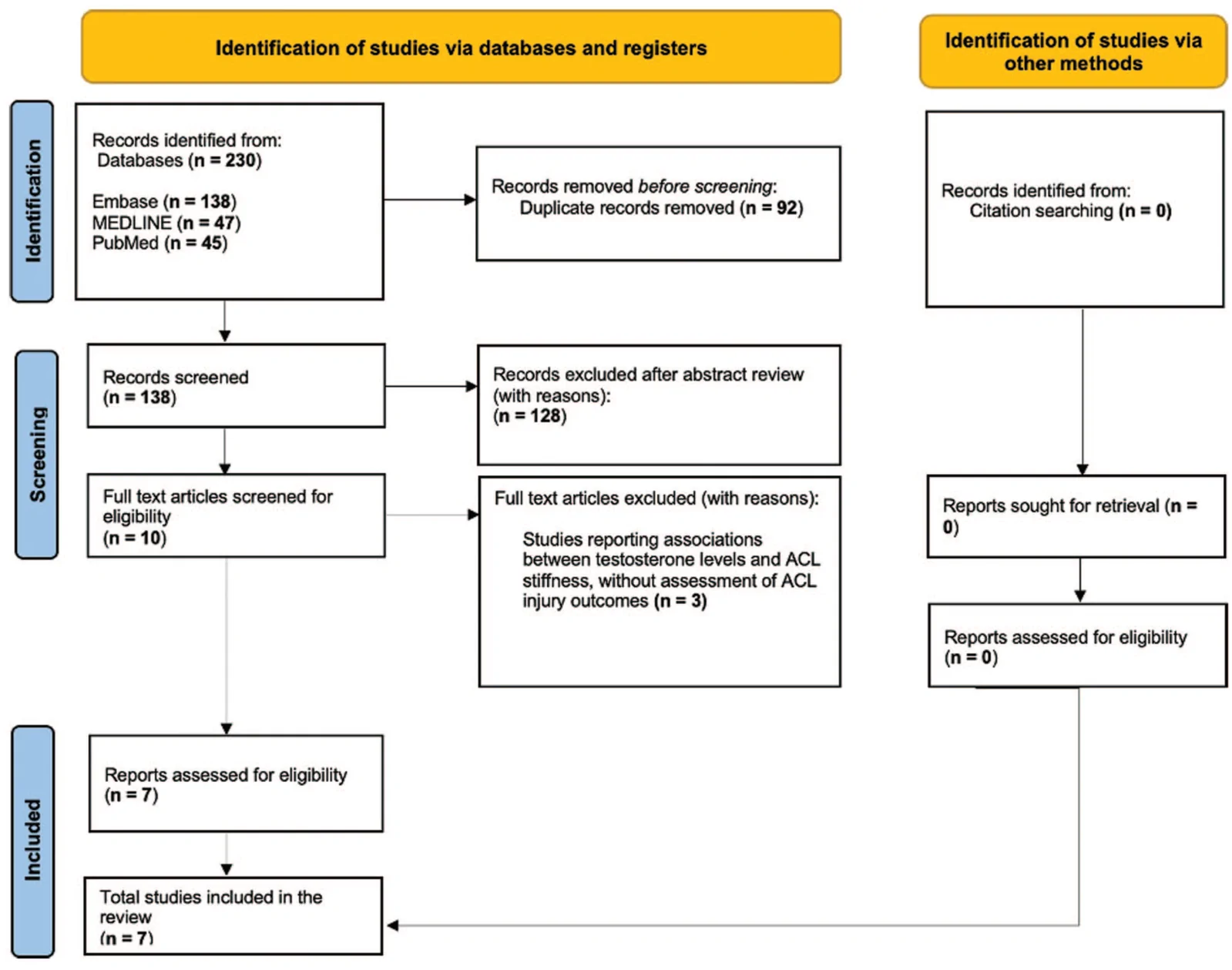
PRISMA flowchart. ACL, anterior cruciate ligament; PRISMA, preferred reporting items for systematic reviews and meta-analyses.

**Table 1.** Summary of all included studies and overall patient demographics

First author (year)	Study design	Level of evidence	Number of patients, N (% male)	Mean age, years ± SD	TRT indication(s)	Outcome assessed
Primary analysis: effect of TRT exposure on (1) ACL tear incidence and (2) ACLR revision, complications, and PROMs						
Brinkman (2025)^6^	Retrospective cohort study	3	Total, 321,678 (87.2%); TRT, 160,839; no TRT, 160,839	TRT, 50.4 ± 12.7; no TRT, 50.4 ± 12.7	Hypogonadism	ACL tear incidence
Quinn (2024)^19^	Retrospective cohort study	3	Total, 851,816 (75.4%); TRT, 425,908; no TRT, 425,908	TRT, median 48; no TRT, median 48	Hypogonadism	ACL tear incidence; ACLR revision
Stickels (2026)^22^	Retrospective cohort study	3	Total, 5282 (86.7%); TRT, 2641; no TRT, 2641	TRT, 40.3; no TRT, 40.8	Hypogonadism	ACLR revision; complications
Wu (2017)^28^	RCT	1	Total, 13 (100%); TRT, 6; no TRT, 7	TRT, 30.4 ± 9.3; no TRT, 26.2 ± 4.1	Counteract muscle atrophy; improve clinical outcomes	Lean mass, strength, PROMs after ACLR
Secondary narrative review: associations between endogenous testosterone levels and (1) ACL tear incidence or (2) ACLR PROMs						
Stijak (2015)^23^	Case-control study	3	Total, 24 (0%); ACL tear, 12; controls, 12	ACL tear, 24.2; control, 24.8	NA	Endogenous testosterone and ACL tear incidence
Stijak (2015)^24^	Case-control study	3	Total 58 (100%); ACL tear, 29; controls, 29	ACL tear, 26.6; control, 27.1	NA	Endogenous testosterone and ACL tear incidence
Thompson (2022)^27^	Prospective cohort study	2	ACLR patients, 20 (100%)	34.0 ± 9.2	NA	Endogenous testosterone levels and PROMs after ACLR

ACL, anterior cruciate ligament; ACLR, ACL reconstruction; NA, not applicable; PROMs, patient-reported outcome measures; RCT, randomized controlled trial; TRT, testosterone replacement therapy.

### Study Demographics

The 7 included studies analyzed collectively 1,178,891 patients, of whom 589,436 received TRT, most commonly prescribed for hypogonadism.

For the primary analysis, 3 large retrospective cohort studies evaluated the association between TRT use and ACL tear incidence.^5,22,26^ Two of these studies also assessed revision rates after ACLR.^22,26^ Across these cohorts, mean age ranged from 40.3 to 50.4 years in TRT users and from 40.8 to 50.4 years in nonusers, with men comprising 75.4% to 87.2% of participants. One RCT evaluated TRT use in patients undergoing ACLR.^33^ This study included a younger male-only cohort (mean age 30.4 years in TRT users and 26.2 years in nonusers) and assessed postoperative changes in lean body mass, muscle strength, and PROMs.

For the secondary narrative review, 2 case-control studies evaluated the association between endogenous testosterone levels and ACL tear incidence.^27,28^ One study included only male participants (n = 58),^28^ while the other ^27^ included female participants (n = 24). Mean age ranged from 24.2 to 26.6 years in ACL tear patients and 24.8 to 27.1 years in controls. One prospective cohort study evaluated the relationship between endogenous testosterone levels and PROMs after ACLR in a cohort of 20 male patients (mean age 34.0 years). ^31^

### Quality of Evidence

The RCT,^33^ assessed using Cochrane RoB2, was judged to have some concerns overall. The study demonstrated low risk of bias across domains related to deviations from intended interventions, missing outcome data, and outcome measurement, but concerns arose from the randomization process and selection of reported results.

The 6 nonrandomized studies were evaluated using MINORS. All studies were classified as moderate quality, with a score of 11 out of a maximum of 16 for the noncomparative study,^31^ and 19 to 21 out of 24 for comparative studies. No study was deemed poor quality (Supplementary Table 2, available online).

### Certainty of Evidence

In the GRADE assessment of the primary analysis, the overall certainty of evidence regarding the association between TRT and ACL tear incidence or ACLR revision and PROMs was low to very low, primarily reflecting the observational study designs and limitations of retrospective database analyses (Table 2). Evidence relating to ACL tear incidence was rated as very low certainty due to risk of residual confounding and lack of detailed clinical variables. Evidence for ACLR revision was rated as very low certainty due to imprecision, limited number of studies, and potential bias arising from heterogeneity in exposure definitions and follow-up durations.

**Table 2.** Summary of findings (GRADE) in the primary analysis: TRT use and ACL tear incidence and ACLR clinical outcomes

Outcome and timepoint	Evidence base	Effect	Certainty of evidence (GRADE)	Reasons for rating*^a^*
ACL tear incidence (2 years)	Two comparative cohorts (meta-analysis)^6,19^	TRT, 0.26% vs non-TRT, 0.10%; OR 2.83, 95% CI, 1.74 to 4.62; *P* < 0.001	Very low (●○○○)	Risk of bias, inconsistency, indirectness
ACL tear incidence (male subgroup)	Two comparative cohorts (meta-analysis)^6,19^	TRT, 0.30% vs non-TRT, 0.11%; OR 2.95, 95% CI, 1.72 to 5.06; *P* < 0.001	Very low (●○○○)	Risk of bias, inconsistency
ACL tear incidence (female subgroup)	Two comparative cohorts (meta-analysis)^6,19^	TRT, 0.15% vs non-TRT, 0.07%; OR 2.06, 95% CI, 1.60 to 2.63; *P* < 0.001	Very low (●○○○)	Risk of bias, imprecision
ACLR revision (2 years)	Two comparative cohorts (meta-analysis)^19,22^	TRT, 6.36% vs non-TRT, 6.21%; OR 1.04, 95% CI, 0.84 to 1.29; *P* = 0.70	Very low (●○○○)	Risk of bias, imprecision
ACLR revision (timing-specific TRT exposure)	One comparative cohort^22^	TRT (≤1 year preoperatively), 17.05% vs 5.17%; OR 3.77, 95% CI, 2.24 to 6.36; *P* < 0.001	Low (●●○○)	Risk of bias, indirectness
Functional outcomes after ACLR (RCT)	One RCT^28^	Increased lean mass at 6 weeks; no difference in KOOS or Tegner	Low (●●○○)	Imprecision (small sample), indirectness

ACL, anterior cruciate ligament; ACLR, ACL reconstruction; GRADE, grading of recommendations, assessment, development and evaluation; KOOS, knee injury and osteoarthritis outcome score; RCT, randomized controlled trial; TRT, testosterone replacement therapy.

a Risk of bias: most included studies are retrospective cohort studies using administrative databases, with potential residual confounding despite matching. Inconsistency: Substantial heterogeneity observed in pooled ACL tear incidence analyses (*I*^ 2^ > 90%). Imprecision: low event rates and wide CIs for several outcomes, particularly ACLR revision. Indirectness: TRT exposure definitions, timing, and dosing varied, and may not reflect physiological testosterone levels. Publication bias: possible due to reliance on large database studies and limited number of included studies.

### Primary Analysis

#### Effects of TRT Exposure on ACL Tear Incidence

ACL tear incidence within 2 years of TRT initiation was pooled from 2 studies,^5,22^ comprising 1,173,494 patients (586,747 TRT users and 586,747 nonusers) (Figure 2). TRT use was associated with a significantly increased risk of ACL tear compared with nonusers (0.26% vs 0.10%; OR, 2.83; 95% CI, 1.74 to 4.62; *P* < 0.001). High statistical heterogeneity was observed (*I*^ 2^ = 95.4%).

**Figure 2.**
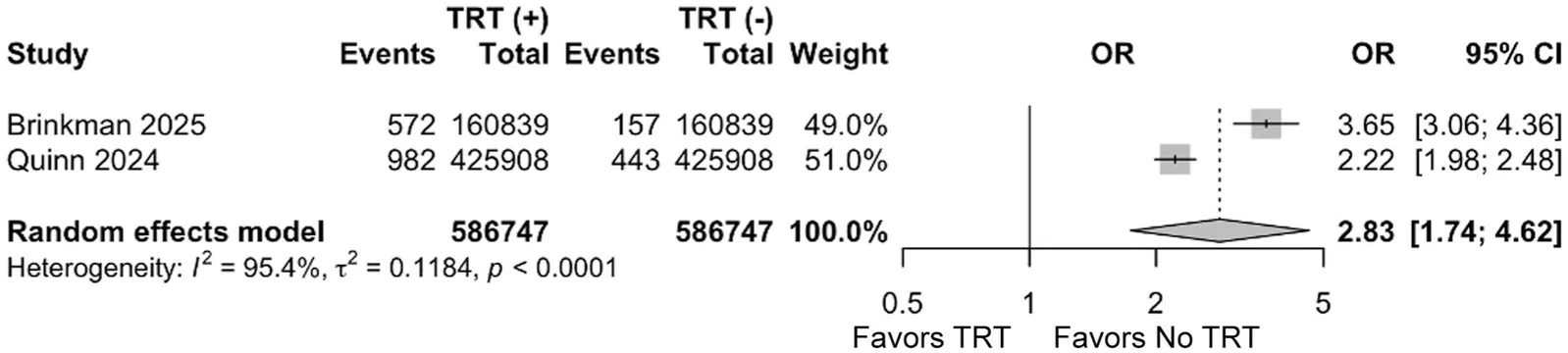
Overall pooled ACL tear incidence within 2 years of TRT initiation. ACL, anterior cruciate ligament; OR, odds ratio; TRT, testosterone replacement therapy.

A subgroup analysis of male patients was conducted across the same 2 studies, including 922,306 men (461,153 TRT users and 461,153 nonusers) (Figure 3). TRT use remained significantly associated with increased ACL tear incidence (0.30% vs 0.11%; OR, 2.95; 95% CI, 1.72 to 5.06; *P* < 0.001). Similar to the overall analysis, substantial heterogeneity was observed (*I*^ 2^ = 95.7%).

**Figure 3.**
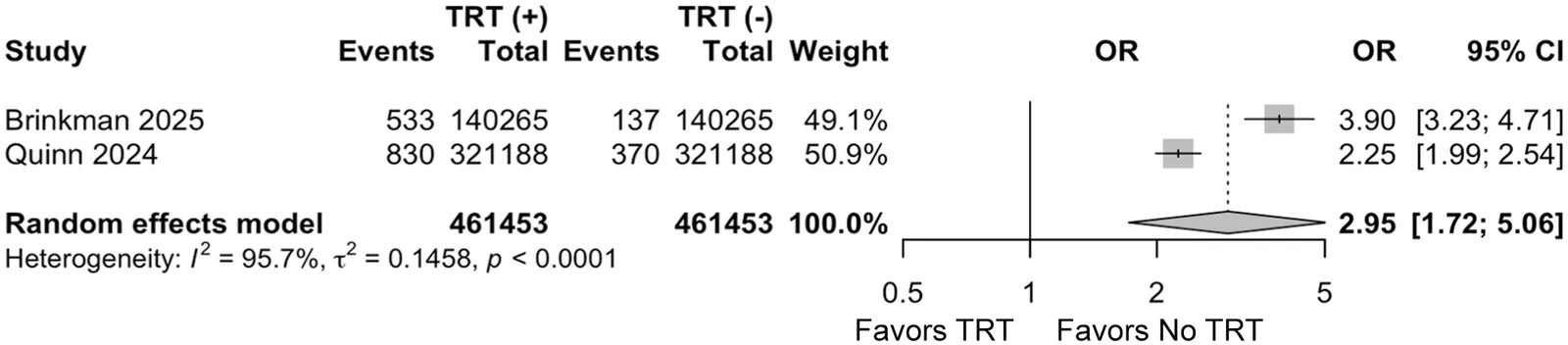
Male-only overall pooled ACL tear incidence within 2 years of TRT initiation. ACL, anterior cruciate ligament; OR, odds ratio; TRT, testosterone replacement therapy.

Subgroup analysis of female patients, comprising 250,588 women (125,294 TRT users and 125,294 nonusers), demonstrated a lower but still statistically significant association between TRT use and ACL tear incidence (0.15% vs 0.07%; OR, 2.06; 95% CI, 1.60 to 2.63; *P* < 0.001), with no heterogeneity (*I*^ 2^ = 0.0%) (Figure 4).

**Figure 4.**
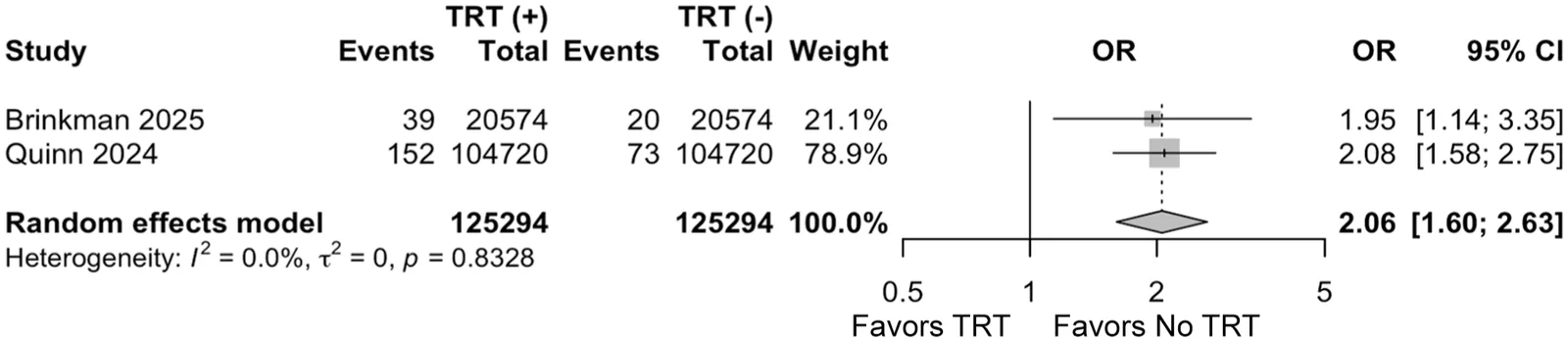
Female-only overall pooled ACL tear incidence within 2 years of TRT initiation. ACL, anterior cruciate ligament; OR, odds ratio; TRT, testosterone replacement therapy.

In 1 study,^5^ age-stratified analysis demonstrated that TRT use was associated with a significantly increased risk of ACL tears across most age groups (25 to 35 years, 36 to 45 years, 46 to 55 years, 56 to 65 years, and >65 years), with OR ranging from 3.91 to 12.3 (*P* < 0.001 to 0.009). The exception was patients <25 years, in whom no significant association was observed (OR, 2.49; 95% CI, 0.44 to 19.25; *P* = 0.32).

#### Effects of TRT Exposure on ACLR Revision and PROMs

Revision rates after ACLR at 2 years follow-up were pooled from 2 studies,^22,26^ comprising 6918 patients (3459 TRT users and 3459 nonusers) (Figure 5). Pooled analysis demonstrated no significant difference in revision rates between TRT users and nonusers (5.26% vs 5.06%; OR, 1.04; 95% CI, 0.84 to 1.29; *P* = 0.70). No statistical heterogeneity was observed (*I*^ 2^ = 0.0%). A notable subgroup analysis from 1 study demonstrated that TRT use within 1 year before primary ACLR was associated with significantly higher revision rates (17.05% vs 5.17%; OR, 3.77; 95% CI, 2.24 to 6.36; *P* < 0.001).^26^ However, when broader TRT exposure windows were considered, including patients with any TRT exposure either before or after surgery, this association was not observed.^26^ No significant differences in postoperative complication rates (infection, deep-vein thrombosis, septic knee, pulmonary embolism) were noted between TRT users and nonusers.^26^

**Figure 5.**
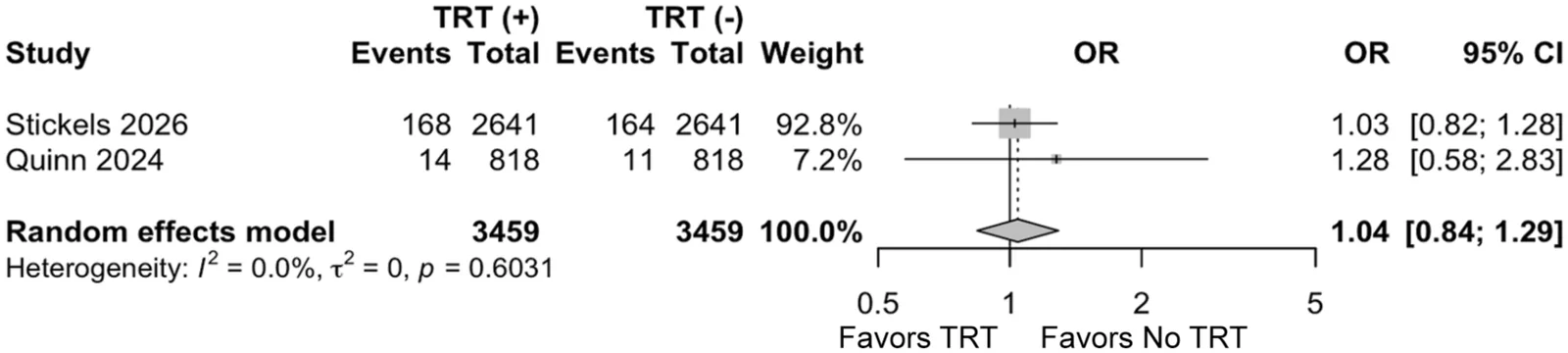
Overall pooled ACLR revision rate with TRT exposure at 2 years follow-up. ACLR, anterior cruciate ligament reconstruction; OR, odds ratio; TRT, testosterone replacement therapy.

One RCT evaluated the effect of TRT on functional outcomes after ACLR.^33^ Patients received 200 mg of intramuscular testosterone weekly for 8 weeks, beginning 2 weeks preoperatively, compared with a placebo saline regimen. At 6 weeks postoperatively, the TRT group demonstrated a significantly greater increase in lean body mass compared with placebo (2.7 ± 1.7 kg vs −0.1 ± 1.5 kg; *P* = 0.01). This difference was not sustained, with no significant differences at 12 (2.2 ± 3.5 kg vs 0.0 ± 1.1 kg; *P* = 0.15) or 24 (2.13 ± 5.30 kg vs 1.05 ± 1.18 kg; *P* = 0.64) weeks . There were no significant differences in extensor strength in the injured leg or functional PROMs (Knee injury and Osteoarthritis Outcome Score [KOOS] scores or Tegner activity scores) between the groups at any timepoint. No adverse events related to TRT administration were reported.

### Secondary Narrative Review

#### Influence of Endogenous Testosterone Levels on ACL Rupture Risk

Two studies evaluated the association between endogenous testosterone levels and ACL injury susceptibility.^27,28^ One of these studies reported that female patients with ACL rupture had significantly lower testosterone levels compared with controls (*P* < 0.01).^27^ In contrast, another study found that male patients with ACL rupture had significantly higher testosterone levels relative to controls (*P* < 0.01).^28^

#### Influence of Endogenous Testosterone Levels on ACLR PROMs

One study evaluated temporal changes in endogenous testosterone levels and their relationship with PROMs after ACLR.^31^ Serum testosterone levels demonstrated an initial postoperative decline, followed by recovery to baseline by 6 weeks, with free testosterone continuing to increase through 12 weeks. This hormonal trajectory paralleled PROMs, which were lowest at 1 week postoperatively and improved subsequently. Specifically, visual analog scale (VAS) pain scores worsened from 0.59 ± 0.9 preoperatively to 2.5 ± 1.8 at week 1, before improving to 0.85 ± 1.1 at week 6 and 0.71 ± 1.0 at week 12. Similarly, Lysholm scores decreased from 70.2 ± 23.6 preoperatively to 43.2 ± 23.7 at week 1, then improved to 75.6 ± 12.8 at week 6 and 84.0 ± 12.3 at week 12. Tegner activity levels followed a comparable pattern, declining from 7.5 ± 2.2 preoperatively to 1.2 ± 1.7 at week 1 and recovering partially to 3.3 ± 2.0 by week 12. Significant differences were observed between preoperative and week 1 Lysholm (*P* = 0.003) and VAS (*P* < 0.001) scores, as well as between week 1 and week 6 Lysholm (*P* < 0.001) and VAS (*P* = 0.006) scores. Although the temporal recovery of testosterone levels mirrored improvements in PROMs, no statistically significant correlations were identified between testosterone and PROMs at any timepoint.

## Discussion

The principal findings of this review are that (1) TRT is associated with significantly increased primary ACL tear incidence within 2 years of initiation; (2) overall, TRT is not associated with 2-year revision rates after ACLR, but there may be increased risk among patients receiving TRT within 1 year before ACLR; and (3) endogenous testosterone levels demonstrate sex-specific associations with ACL rupture risk.

In the primary analysis, an association between TRT use and increased 2-year ACL tear incidence was found. This aligns with orthopaedic literature, demonstrating increased rates of musculoskeletal injury with TRT use, such as at the distal biceps, quadriceps, and rotator cuff.^18,23,30^ The exact mechanism by which TRT influences ACL integrity is unknown. Potentially, this effect may be underpinned by testosterone modulating connective tissue synthesis and remodeling, thereby altering tensile properties.^1^ Also, aromatization of exogenous testosterone to estrogen may affect collagen cross-linking adversely by reducing lysyl oxidase activity, inadvertently weakening ligament structure.^14^

However, the association between TRT and ACL tear incidence is multifactorial, not solely biologically mediated. Alternative explanations involve behavioral factors. For instance, testosterone is thought to promote motivation and effortful behavior through modulating neural dopamine pathways.^21^ Hence, those undergoing TRT may have increased motivation to engage in physical activity, such as sport, which may predispose them to ACL injury.^1^

Furthermore, TRT was not associated with overall 2-year revision rates after ACLR, but 1 subgroup demonstrated increased revision risk if TRT use was within 1 year before surgery.^26^ This apparent discrepancy may reflect differences between native ACL injury and graft failure. Primary ACL tears may be influenced by hormone-related changes in ligament properties and exposure to high-risk activity, whereas graft failure is multifactorial and commonly associated with recurrent trauma, technical factors such as femoral tunnel malposition, and return to high-level activity.^7,32^ These competing determinants, together with the limited evidence base, may obscure any independent effect of TRT.

The secondary narrative review highlights the sex-specific role of endogenous testosterone in ACL injury risk. Women with ACL rupture demonstrated lower testosterone levels, while higher levels were found in men with a ruptured ACL.^27,28^ This implies the relationship between testosterone and ACL injury is not linear, but instead the product of a balance between testosterone and estrogen. Previous work has demonstrated that the ratio of estrogen to testosterone influences ACL stiffness, with higher testosterone levels associated with increased stiffness and higher estrogen levels associated with reduced stiffness^15^; also, female ACLs are thought to be more sensitive to hormonal fluctuations, potentially due to sexually dimorphic receptor densities.^3^ This interplay may partly explain the divergent findings observed between sexes and underscores the importance of considering endocrine balance perioperatively. Similarly, in ACLR, higher testosterone does not appear to adversely affect strength or PROMs,^31^ with the exact mechanisms unknown. Potentially, higher testosterone leads to increased strength recovery postoperatively, which could improve both neuromuscular performance and function, while mitigating pain, as reflected by PROMs.^31^

The findings of this review have important clinical implications. Clinicians should be aware of a potential association between TRT and ACL tear risk. Despite this, TRT does not seem to adversely affect ACLR revision or PROMs. Thus, there is currently insufficient evidence to recommend discontinuing TRT before ACLR, especially given its potential benefits for lean muscle mass. Instead, a patient-specific approach is essential, involving assessment of hormonal status, activity level, patient goals, and individual risk factors. Patients should be counseled on the potential trade-off between enhanced neuromuscular performance and possible alterations in ligament integrity, particularly when engaging in high-risk athletic activity.

Several limitations of this study should be considered. The evidence base was small, with only 2 studies contributing to each meta-analysis, resulting in imprecision and limited statistical power. Most included studies were retrospective database cohorts, introducing risks of coding error, misclassification, and residual confounding despite propensity matching. Key determinants of ACL injury risk, including activity level, sport participation, types of sporting activities, and neuromuscular function, were not captured, limiting causal inference. There was substantial heterogeneity in TRT definitions, including variability in timing, duration, and dosing, with limited serum testosterone level data, precluding assessment of dose dependent effects and contributing to high statistical heterogeneity. Study populations were older and predominantly male, limiting generalizability to younger mixed athletic cohorts. Evidence on endogenous testosterone was limited, heterogeneous, and inconsistent. The inability to distinguish physiological from supraphysiological testosterone exposure represents a key limitation, as these interventions likely exert different biological effects. Outcome reporting was also inconsistent, with limited data on long term function and graft integrity, contributing to the low to very low certainty of evidence.

Future research should aim to clarify the relationship between testosterone and ACL injury through prospective studies incorporating detailed hormonal profiling, activity metrics, and biomechanical assessment. Studies differentiating physiological TRT from supraphysiological exposure are particularly needed. In addition, mechanistic studies integrating imaging, molecular biology, and biomechanical testing may provide further insight into the effects of testosterone on ACL structure and function. A more comprehensive understanding of these relationships may enable targeted strategies for ACL injury prevention and perioperative hormonal optimization in patients undergoing ACLR.

## Conclusion

TRT is associated with an increased risk of ACL tear within 2 years of initiation but does not seem to influence 2-year revision rates after ACLR. The evidence suggests endogenous testosterone may affect tear susceptibility in a sex-dependent manner, without adversely impacting postoperative ACLR PROMs, strength, and complications. However, the overall certainty of evidence remains low, and the observed association is likely multifactorial. Further prospective studies are required to clarify the clinical significance of testosterone in ACL injury risk and postoperative recovery after ACLR.

## Supplemental Material

sj-docx-1-sph-10.1177_19417381261486660 – Supplemental material for Testosterone and Anterior Cruciate Ligament Injury and Reconstruction Outcomes: A Systematic Review and Meta-analysis Supplemental material, sj-docx-1-sph-10.1177_19417381261486660 for Testosterone and Anterior Cruciate Ligament Injury and Reconstruction Outcomes: A Systematic Review and Meta-analysis by Dave Osinachukwu Duru, David Kwan Ryung Lee, Prushoth Vivekanantha, Marc Daniel Bouchard, Osama Alzobi, Loiy Alkhatib, Bashir A. Zikria, Ashraf Hantouly and Moin Khan in Sports Health

sj-docx-2-sph-10.1177_19417381261486660 – Supplemental material for Testosterone and Anterior Cruciate Ligament Injury and Reconstruction Outcomes: A Systematic Review and Meta-analysis Supplemental material, sj-docx-2-sph-10.1177_19417381261486660 for Testosterone and Anterior Cruciate Ligament Injury and Reconstruction Outcomes: A Systematic Review and Meta-analysis by Dave Osinachukwu Duru, David Kwan Ryung Lee, Prushoth Vivekanantha, Marc Daniel Bouchard, Osama Alzobi, Loiy Alkhatib, Bashir A. Zikria, Ashraf Hantouly and Moin Khan in Sports Health
