# Supplementary material for: Testosterone and Anterior Cruciate Ligament Injury and Reconstruction Outcomes: A Systematic Review and Meta-analysis
Source: Sports Health. 2026 Oct 15:19417381261486660. Online ahead of print. doi: 10.1177/19417381261486660 (PMC13675417; doi:10.1177/19417381261486660)
Supplement: Supplementary file 1 [file sj-docx-1-sph-10.1177_19417381261486660.docx]

**Supplementary Table 1. Search strategies**

**Database: MEDLINE (via Ovid) <1946 to 2026 March 14>**

1. (“testosterone” or “androgen*” or hypogonad* or "low testosterone" or "testosterone deficiency" or "testosterone level*" or "serum testosterone" or "total testosterone" or "free testosterone" or "testosterone replacement" or "testosterone replacement therapy" or TRT or "androgen replacement" or "androgen therapy" or "anabolic steroid*" or AAS or “dihydrotestosterone” or “DHT”).mp. (232654)
2. exp testosterone/ or exp androgen/ or exp hypogonadism/ or exp anabolic steroid/ (119103)
3. 1 or 2 (239017)
4. ("anterior cruciate ligament" or ACL or "ACL rupture" or "ACL tear*" or "ACL injur*" or "anterior cruciate ligament injur*" or "anterior cruciate ligament rupture" or "anterior cruciate ligament tear*" or "ACL reconstruction" or ACLR or "revision ACL reconstruction").mp. (38504)
5. 3 and 4 (47)

**Database: Embase (via Ovid) <1974 to 2026 March 14>**

1. (“testosterone” or “androgen*” or hypogonad* or "low testosterone" or "testosterone deficiency" or "testosterone level*" or "serum testosterone" or "total testosterone" or "free testosterone" or "testosterone replacement" or "testosterone replacement therapy" or TRT or "androgen replacement" or "androgen therapy" or "anabolic steroid*" or AAS or “dihydrotestosterone” or “DHT”).mp. (343821)
2. exp testosterone/ or exp androgen/ or exp hypogonadism/ or exp anabolic steroid/ (308842)
3. 1 or 2 (431918)
4. ("anterior cruciate ligament" or ACL or "ACL rupture" or "ACL tear*" or "ACL injur*" or "anterior cruciate ligament injur*" or "anterior cruciate ligament rupture" or "anterior cruciate ligament tear*" or "ACL reconstruction" or ACLR or "revision ACL reconstruction").mp. (53000)
5. 3 and 4 (138)

**Database: PubMed <from inception to 2026 March 14>**

1. ("testosterone"[tiab] OR androgen*[tiab] OR hypogonad*[tiab] OR "low testosterone"[tiab] OR "testosterone deficiency"[tiab] OR "testosterone level*"[tiab] OR "serum testosterone"[tiab] OR "total testosterone"[tiab] OR "free testosterone"[tiab] OR "testosterone replacement"[tiab] OR "testosterone replacement therapy"[tiab] OR TRT[tiab] OR "androgen replacement"[tiab] OR "androgen therapy"[tiab] OR "anabolic steroid*"[tiab] OR AAS[tiab] OR "androgenic anabolic steroid*"[tiab] OR dihydrotestosterone[tiab] OR DHT[tiab] OR "androgen deprivation"[tiab] OR "androgen suppression"[tiab] OR "Testosterone"[Mesh] OR "Androgens"[Mesh] OR "Hypogonadism"[Mesh] OR "Anabolic Agents"[Mesh]) (233500)
2. ("anterior cruciate ligament"[tiab] OR ACL[tiab] OR "ACL tear*"[tiab] OR "ACL rupture"[tiab] OR "ACL injur*"[tiab] OR "anterior cruciate ligament tear*"[tiab] OR "anterior cruciate ligament rupture"[tiab] OR "anterior cruciate ligament injur*"[tiab] OR "ACL reconstruction"[tiab] OR ACLR[tiab] OR "revision ACL reconstruction"[tiab] OR "Anterior Cruciate Ligament"[Mesh] OR "Anterior Cruciate Ligament Injuries"[Mesh] OR "Anterior Cruciate Ligament Reconstruction"[Mesh]) (38586)
3. #1 AND #2 (45)
