# Supplementary material for: Testosterone and Anterior Cruciate Ligament Injury and Reconstruction Outcomes: A Systematic Review and Meta-analysis
Source: Sports Health. 2026 Oct 15:19417381261486660. Online ahead of print. doi: 10.1177/19417381261486660 (PMC13675417; doi:10.1177/19417381261486660)
Supplement: Supplementary file 2 [file sj-docx-2-sph-10.1177_19417381261486660.docx]

**Supplementary Table 2. MINORS quality assessment of all included papers in the review.**

|  | **Criteria** | | | | | | | | | | | | | | |
| --- | --- | --- | --- | --- | --- | --- | --- | --- | --- | --- | --- | --- | --- | --- | --- |
| **References** | **1** | **2** | **3** | **4** | **5** | **6** | **7** | **8** |  | **9** | **10** | **11** | **12** | **TOTAL MINORS SCORE** | **Maximum possible score** |
| Brinkman et al., 2025 ^6^ | 2 | 1 | 0 | 2 | 2 | 2 | 2 | 0 |  | 2 | 2 | 2 | 2 | 19 | 24 |
| Quinn et al., 2024 ^19^ | 2 | 1 | 0 | 2 | 2 | 2 | 2 | 2 |  | 2 | 2 | 2 | 2 | 21 | 24 |
| Stickels et al., 2026 ^22^ | 2 | 1 | 0 | 2 | 2 | 2 | 2 | 1 |  | 2 | 2 | 2 | 2 | 20 | 24 |
| Stijak et al., 2015a ^24^ | 2 | 1 | 1 | 2 | 2 | 1 | 2 | 1 |  | 2 | 2 | 2 | 2 | 20 | 24 |
| Stijak et al., 2015b ^23^ | 2 | 1 | 1 | 2 | 2 | 1 | 2 | 2 |  | 2 | 2 | 2 | 2 | 21 | 24 |
| Thompson et al., 2022 ^27^ | 2 | 1 | 2 | 2 | 1 | 1 | 2 | 0 |  |  |  |  |  | 11 | 16 |

*Note:* MINORS = Methodological Index for Non-randomized Studies

1. A clearly stated aim

2. Inclusion of consecutive patients

3. Prospective collection of data

4. Endpoints appropriate to the aim of study

5. Unbiased assessment of study endpoint

6. Follow-up period appropriate for the aim of study

7. Loss to follow-up less than 5%

8. Prospective calculation of study size

9. An adequate No TRT group

10. Contemporary groups

11. Baseline equivalence of groups

12. Adequate statistical analyses
